# Antibacterial Efficacy and Healing Potential of Honey from Different Zones in Nigeria on Diabetic-Induced Wound Infection in Wistar Rats

**DOI:** 10.1155/2022/5308435

**Published:** 2022-10-21

**Authors:** Obakpororo Ejiro Agbagwa, Chimezie Ekeke, Precious Chidinma Israel

**Affiliations:** ^1^Department of Microbiology, Faculty of Science, University of Port Harcourt, Port Harcourt, Rivers State, Nigeria; ^2^Department of Plant Science, Faculty of Science, University of Port Harcourt, Port Harcourt, Rivers State, Nigeria

## Abstract

There is an increase in drug-resistant strains causing infection, thus making available therapeutics less effective. As resistance increases, modern medicine focuses on the antibacterial potential of natural products, which can aid in wound healing. The present study determined Nigeria honey's antibacterial efficacy in treating diabetes-induced wound infections in Wistar albino rats. 54 Wistar rats randomly divided into 9 groups of 6 each were used for the study: group I (negative control, no treatment), group II (positive control, diabetes without treatment), group III (diabetes treated with 1% silver sulfadiazine), and groups IV–IX (diabetes treated with different honey samples). Physiochemical analysis and microbiological and antibacterial activity of the honey samples were determined. The treatments were carried out for 17 days, and wound contraction, malondialdehyde (MDA) levels, and catalase activity were measured. Results obtained showed that the most effective honey was DCH (21.5 ± 2.12), followed by HBP + M (15 ± 2.12) and TRB, JS, and HBP (13 ± 2.8; 13 ± 1.41; 13.5 ± 0.71) for antibacterial activity on *Staphylococcus aureus*. Microbiologically, no coliform was detected in all the samples, confirming the honey's quality. The amount of lipid peroxidation was raised in the diabetic group with no treatment, 1% silver sulfadiazine group, and JS group, while no significant reduction was observed in other groups. Differences in wound contraction were significantly notable on various days of measurement, day 3 (*p* < 0.002), day 6 (*p* < 0.046), and day 9 (*p* = 0.00). The catalase level in the different treatment groups increased significantly (*p* < 0.05), implying an antioxidant potential of the different honey samples except for Jos honey. The study concludes that honey infused with moringa was faster and more efficient in healing diabetic wounds than other honey samples and silver sulfadiazine.

## 1. Introduction

Diabetes is a severe metabolic disorder that causes physiological changes in tissues and cells, leading to diabetic wounds in the lower extremities in some patients. Diabetes can cause bacterial infection and microbial resistance complications, leading to wound healing and amputation delays. The need for alternative antimicrobial techniques has increased the interest in the therapeutic use of traditional medicines such as plants and plant-based products such as honey, ginger, garlic, and moringa. Plant extract-derived products have been shown to promote wound healing by lowering inflammatory cell influx in situ, boosting angiogenesis and fibroblastic proliferation, or speeding up reepithelization. They also encourage a combination of different pathological actions [1]. Honey is a natural product of bees produced from the nectar of plants. It is rich in several phenolic compounds, enzymes, and sugars with antioxidant, anticarcinogenic, anti-inflammatory, and antimicrobial potential.

Research has led to the development of honey-based adhesives for wound care and other biomedical applications. Honey is helpful in wound healing because it can accelerate dermal repair and epithelialization, promote angiogenesis and immune response, and reduce healing-related infections with pathogenic microorganisms [2]. Microbiological analysis of honey is carried out to ascertain the sanitary and quality of the honey. Microorganisms found in honey are usually dormant and limited in number. The organisms in honey are from the bees, while others are introduced during production, such as handling procedures [3, 4]. These drug-resistant strains are also implicated in wound infections, making available therapeutics nonfunctional or less effective. This has led to the focus on natural products with antioxidant potential, such as honey and other plant-based products, which have been used since ancient times to treat various ailments based on their antimicrobial potential. Honey also has anti-inflammatory potency, antioxidant potential, and free radical scavenging ability and is an immunomodulator [5–7]. There has been a global rise in antibiotic-resistant bacteria; thus, there is a need for a continued search for affordable agents with speed and no side effects for treating ailments. Therapeutic agents that are plant-based with bioactive components are used for treatments because they lessen and eliminate chronic microbial infection and inflammation by demonstrating anti-inflammatory and antioxidant properties [8]. Honey is rich in compounds such as methylglyoxal, hydrogen peroxide, phenolics, and bee defensin-1 peptide. It has low moisture content and high osmolarity and acidity. The unique composition of honey and its intrinsic characteristics has directly affected the growth and survival of bacteria [9–11]. Wang et al. [12] showed that honey has broad-spectrum bactericidal properties, aids in managing wound infection, enhances the proliferation of the epithelium, and absorbs oedema around the wound. According to previous studies, honey can reduce the activity of cyclooxygenases 1 and 2 (COX1 and COX2) that intervene in the synthesis of prostaglandins involved in inflammatory responses [13, 14]. Some side effects are traditionally associated with using honey in people with diabetes, which can be controlled based on a doctor's advice [15]. Contemporary medicine focuses on natural, unconventional, and nonantibiotic treatments with antimicrobial activity, antioxidant and anti-inflammatory properties, and therapeutic purposes [16–18]. Therefore, the present study evaluated the antibacterial and in vivo efficacy of honey and honey infused with some plant extracts in promoting wound healing in diabetes-induced wound infection in Wistar rats.

## 2. Materials and Methods

### 2.1. Collection of Honey Samples

Eleven honey samples were used for this study. These honey were obtained from different beehive keepers in different geographical regions of Nigeria, namely, Nsukka south-east (NSK), Kaduna north-western (KAD), Taraba north-eastern (TRB), Jos north-central (JS), Obudu south-south (OBD), Imo south-east (IMS), Lagos south-west (DCH), US grade A branded honey (KL), honey bee pure honey (HBP), honey bee pure honey infused with moringa (HBP + M), and honey bee pure honey infused garlic (HBP + G). All samples were kept at room temperature with tight-fitting lids.

### 2.2. Microbiological Analysis

This research was conducted at the University of Port Harcourt's Medical Microbiology Laboratory. *Staphylococcus aureus* stock isolates from the wound were acquired from the Medical Microbiology and Parasitology Laboratory at the University of Port Harcourt Teaching Hospital in Rivers State, Nigeria. *Staphylococcus aureus* isolates were reidentified by cultural and biochemical methods. In brief, a colony was picked from the nutrient agar slant containing *Staphylococcus aureus* obtained from the culture bank and streaked onto mannitol salt agar (MSA). This was incubated at 37°C for 24 hours, and characteristic *Staphylococcus aureus* was purified. Identification was based on colony characteristics, Gram reaction, catalase, and coagulase test.

#### 2.2.1. Microbial Population and Preparation of Honey Concentrations

The spread plate technique was used to determine the microbial population of honey samples. Honey samples were diluted in sterile distilled water, and different concentrations of honey were generated in 70, 50, 20, and 10%, and plates were incubated for 24 hours at 37°C [19].

#### 2.2.2. Determination of the Antibacterial Activity of Honey

The agar well diffusion technique was used to evaluate susceptibility [20]. The inoculums were made in sterile normal saline and compared to 0.5 McFarland standard (1 × 10^8^ CFU/ml) for opacity. A sterile swab was dipped into the isolate solution, pressed clear of excess fluid against the edge of the tube, and then distributed over the agar plate. The test organism was evenly distributed on the Mueller-Hinton agar (Oxoid) surface, and the plates were left on the bench to absorb the excess moisture. Wells were formed in the agar medium with a sterile cork borer (6-millimeter (mm) diameter), and 0.1 ml of honey in various concentrations was applied to the wells on the plate. The plates were incubated for 24 hours at 37°C. The inhibition's mean diameters were measured in mm.

### 2.3. Physiochemical Analysis of Selected Honey Samples

Selected physicochemical parameters such as pH, moisture content, ash, fibre, crude protein, fructose, glucose, sucrose, tannin, phenol, and flavonoid were determined using the methods of Agbagwa [21].

### 2.4. Study Design/Experimental Animals

Adult albino rats (120.4–300.5 g) with a mean weight of 185.4 g were housed in a conventional and regulated laboratory environment with a 12 : 12 h dark and light cycle to acclimatize. The rats were fed on rodent laboratory chow and had access to water and *ad libitum*. Before the start of the investigation, the vital parameters of the animals, including temperature, weight, and fasting blood glucose level, were evaluated and determined to be consistent with excellent health [18]. The 54 rats were randomly separated into 9 groups (*n* = 6) and distinguished with dyes on the head, neck, midbody, tail, and right leg and no dye. The 9 experimental groups formed are presented in Table 1.

### 2.5. Induction of Diabetes

Diabetes was developed in 48 Wistar rats after 14 days of acclimation by intraperitoneal injection (150 mg/kg body weight) of freshly produced alloxan monohydrate (150 mg/kg; purity >98%; Sigma-Aldrich, USA) in normal saline after overnight fasting [22]. Fasting glucose levels were measured from tail-vein blood 48 hours after alloxan administration using a glucose autoanalyzer (ACCU-CHEK Active® Blood Glucose Monitoring System, Mannheim, Germany). Forty-eight rats with constant blood glucose levels over 200 mg/dL (11.1 mmol/L) were classified as diabetic, and animals were chosen for wound formation [23].

### 2.6. Wound Creation

The experimental animals' dorsal skin was shaved and swabbed with ethanol under anaesthesia with an intramuscular injection of 5% ketamine and 2% xylazine. Following anaesthetic recovery, the experimental animals were placed in disinfected cages in groups for continued wound monitoring. An entire thickness punch biopsy wound was produced on the dorsum of each rat, and each wound was infected with 1 × 10^8^ CFU/ml of *Staphylococcus aureus* prepared for the purpose.

### 2.7. Confirmation of Infection/Topical Application of Topical Antibacterial Agents

The wounds were left untreated for 24 hours before infection. Additionally, swab samples of wound exudate were taken and cultured on mannitol salt agar plates and were verified using Gram reaction, catalase, and coagulase test [24]. Following the confirmation of wound infection, 1% silver sulfadiazine (SS) (manufactured by Salutas Pharma GmbH 39171 Osterweddingen, Germany) and 0.1 ml honey samples were applied to the wound surface to form a thin layer completely covering the wound. Wounds on rats in both the negative and positive control groups were left untreated [25]. The effect of topical application on healing in dorsal wounds in alloxan-induced diabetic rats was investigated on different days for wound contraction and swabbed for bacterial clearance compared with the typical SS as the reference cream due to its antibacterial activity.

### 2.8. Assessment of Significant Markers

#### 2.8.1. Gross Wound Assessment, Wound Contraction Measurement, and Evaluation of Wound Bacterial Clearance

Each animal was evaluated for gross wound healing indices (wound surface exudation, edge oedema, surface hyperemia, granulation tissue, and rate of wound contraction) [18]. The reduction in the wound area was measured each day by marking the area using the meter rule, and a picture of each wound was taken. Wound contraction was expressed as a percentage of the healed wound area:

(1) $$\begin{array}{c} \text{percentage of wound closure}\left(\%\right)=\frac{\text{wound area on day }0-\text{wound area on day }9\text{th}}{\text{the initial area of the wound}}\times 100. \end{array}$$

On days 3, 5, and 7, swabs were taken from the wound exudates for bacterial culture and colony quantification.

#### 2.8.2. Determination of Biochemical Parameters (Blood Glucose, Lipid Peroxidation, and Antioxidant)

The glucose level was tested to confirm diabetes induction. Blood samples were taken, and the blood glucose level was determined using a glucometer (ACCU-CHEK Active®). The TBA reaction was used to assess the quantity of malondialdehyde (MDA) in wound tissue and tissue homogenates to determine the rate of lipid peroxidation. The reaction of MDA with thiobarbituric acid (TBA) in acidic circumstances and at higher temperatures results in a pink MDA-(TBA)_2_ complex that can be detected spectrophotometrically at 532 nm. Tissue homogenate (10%) of granulation tissues was produced in 0.02 M phosphate-buffered saline and used to calculate catalase (CAT). The sample was combined in a test tube containing tissue homogenate (0.1 ml) and hydrogen peroxide (0.5 ml) and incubated in a water bath for 60 seconds at 37°C before being measured using a spectrophotometer at 480 nm [18, 26].

### 2.9. Quality Control

SOP was followed to assure data quality at all stages of the investigation (preanalytical, analytical, and postanalytical). The culture media were produced following the manufacturer's instructions.

### 2.10. Statistical Analysis

The results were statistically evaluated following double sampling, and the mean values and standard deviations were given. One-way ANOVA was employed to examine the differences across groups, and *p* < 0.05 was considered significant [27]. One sample *t*-test was used to compare the sample mean to the population mean.

## 3. Results

### 3.1. Microbiological Analysis of Selected Honey

Total viable count (TVC) and total coliform count (TCC) were carried out on the selected honey samples to ascertain the microbiological quality of the samples. Total coliform counts were not detected in all the honey samples studied, and TVC ranged from 3.5 × 10^3^ to 7.3 × 10^4^ CFU/ml. The least TVC was observed in honey sample KL and the highest in NSK, as shown in Table 2.

### 3.2. Antibacterial Efficacy of Honey and Effect on *Staphylococcus aureus* Population

**Figure 1:**
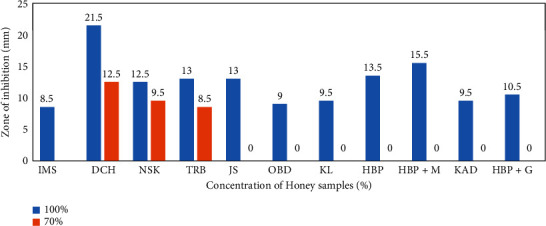
Antibacterial efficacy of selected honey on *Staphylococcus aureus*.

**Figure 2:**
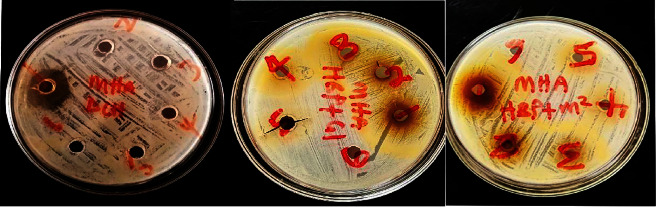
Zone of inhibition of honey on *Staphylococcus aureus*.

The effect of selected honey samples on *Staphylococcus aureus* was determined by the well-in-agar method at different concentrations (Figure 1). Of the 11 honey samples studied, only 3 (DCH 21.5 mm ± 0.71; NSK 9.5 mm ± 0.71; TRB 8.5 mm ± 2.12) showed a zone of inhibition at 70%. The others were effective when used undiluted (100%). The most effective honey was DCH (21.5 mm ± 2.12), followed by HBP + M (15 mm ± 2.12) and TRB, JS, and HBP (13 mm ± 2.8; 13 mm ± 1.41; 13.5 mm ± 0.71).  Figure 2 presents the image of the zones of inhibitions in some of the honey samples.

### 3.3. Physiochemical Parameters of Selected Honey Samples

The results obtained for the physical and chemical parameters of the honey samples are shown in Table 3. The pH of the selected samples ranged from 3.9–4.7, moisture content 12.16–27.43%, ash content 0.04–0.26, crude 0.41–1.72%, lipid 0.2–2.84%, degree Brix 72–83.4, fructose 25.69–38.67%, glucose 25.41–39.1%, sucrose 1.73–2.13%, tannin 1.5–6.88%, phenol 156.7–263.1 mg/100 g, and flavonoids 49.83–71.31%. The fibre was not detected in all the honey samples. The average moisture content of the honey samples was below 20% for HBP, HBP + M, OBD, DCH, KAD, KL, JS, NSK, and IMS honey. Sugar analysis of all the honey samples showed that the fructose contents varied, averaging 31.38 ± 0.76%. The highest fructose content was seen in TRB (37.39%) and the least in IMS (25.69%). The mean glucose content was 30.07 ± 0.31%. The highest glucose content was seen in IMS (39.1%) and the lowest in TRB and NSK (25.41%), while the mean value for sucrose was 1.88 ± 0.05.

### 3.4. Effect of Honey Treatment on Wound Contraction

**Figure 3:**
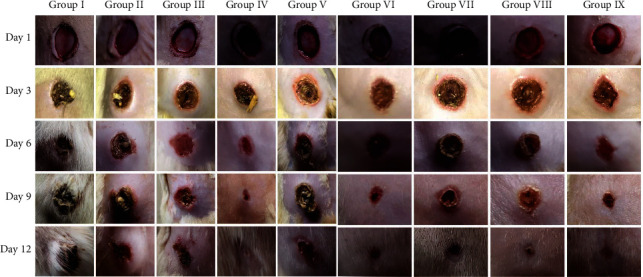
Image of wound contraction in experimental groups for days 1, 3, 6, 9, and 12.

**Figure 4:**
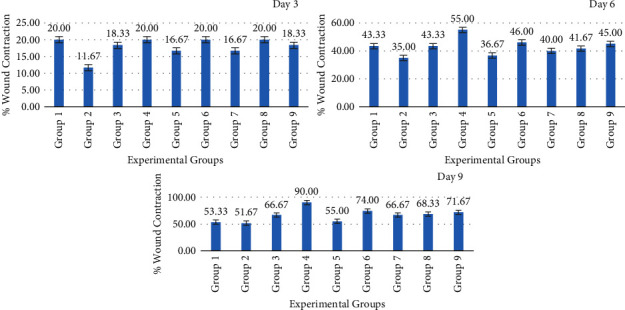
Wound contraction in experimental groups on days 3, 6, and 9 after wounding.

Similar to silver sulfadiazine treatment, honey generated a time-dependent increase in wound contraction compared to the control groups, as seen by wound photographs taken on different days (Figure 3). The percentage of mean wound contraction in the silver sulfadiazine and honey-treated groups was substantially higher than that in the positive control group (diabetic control) on days 3, 6, and 9 of the treatment (*p* > 0.05) (Figure 4). At day 3, the mean size of experimental animal wounds was 18.33 ± 4.08%, 20.00 ± 0.00%, 16.67 ± 5.16%, 20.00 ± 0.00%, 16.67 ± 5.16%, 20.00 ± 0.00%, and 18.33 ± 4.08% for groups 3, 4, 5, 6, 7, 8, and 9, respectively, which was substantially reduced to 43.33 ± 8.17%, 55.00 ± 5.48%, 36.67 ± 5.16%, 46.00 ± 8.94%, 40.00 ± 12.65%, 41.67 ± 17.22%, and 45.00 ± 8.37%, respectively, after three days of treatment, and wounds were gradually reduced to 66.67 ± 16.33%, 90.00 ± 0.00%, 55.00 ± 10.49%, 74.00 ± 11.40%, 66.67 ± 8.17%, 68.33 ± 7.52%, and 71.67 ± 7.52%, respectively, three days of treatment. Differences in the wound diameter (a reflection of wound contraction) were significantly notable on various days of measurement, day 3 (*p* < 0.002), day 6 (*p* < 0.046), and day 9 (*p*=0.00).

#### 3.4.1. Effect of Honey on Wound Surface Exudation (Wetness/Dryness)

**Figure 5:**
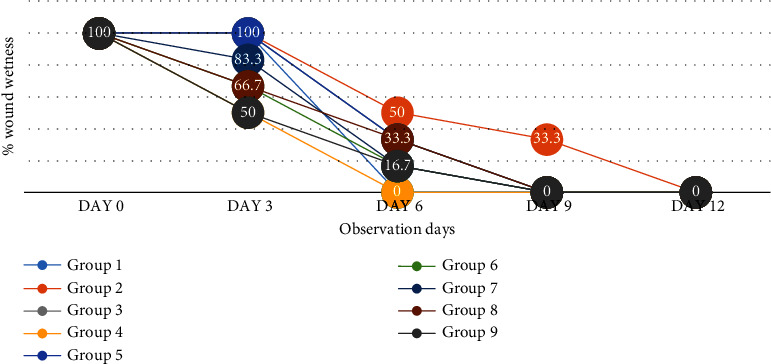
Percentage of wound exudation in experimental groups on days 3, 6, 9, and 12 after wounding.

All groups had significant wound exudation (Figure 5), which diminished gradually from day 0 to day 12. It was completely missing in the HBP + M and nondiabetic control groups, and it was less prevalent in the SS (33.3%), JS (33.3%), NSK (16.7%), TRB (16.7%), DCH (33.3%), and KAD (16.7%) groups with the trend being NSK, TRB, KAD < JS, SS, and DCH compared to the diabetic control group at day 6 (*p* > 0.05).

#### 3.4.2. Effect of Honey on Wound Edge Oedema, Wound Hyperemia (Colour), and Granulation Tissue

**Figure 6:**
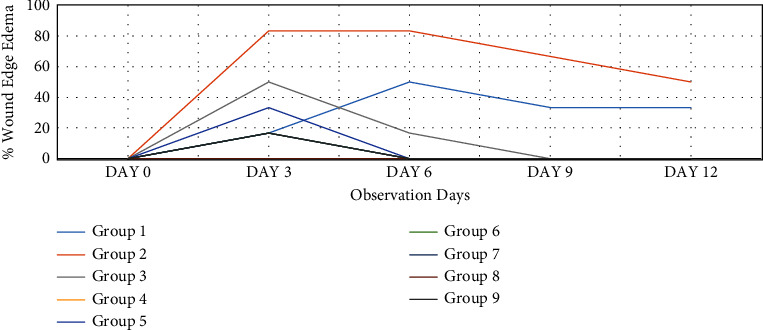
Wound edge oedema (%) in experimental groups on days 3, 6, 9, and 12 after wounding.

**Figure 7:**
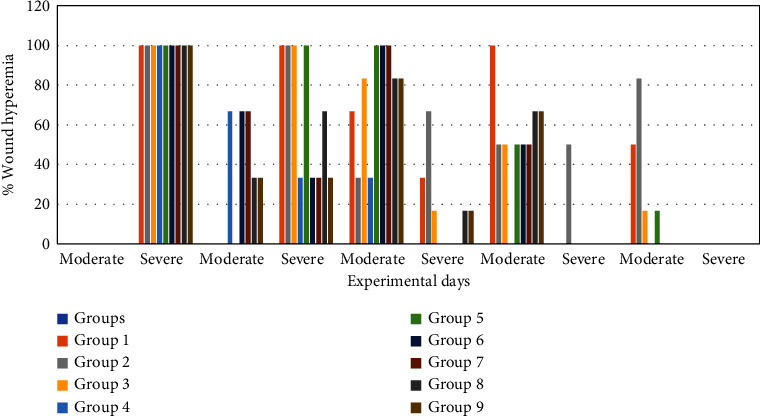
Wound hyperemia (%) in experimental groups on days 3, 6, 9, and 12 after wounding.

**Figure 8:**
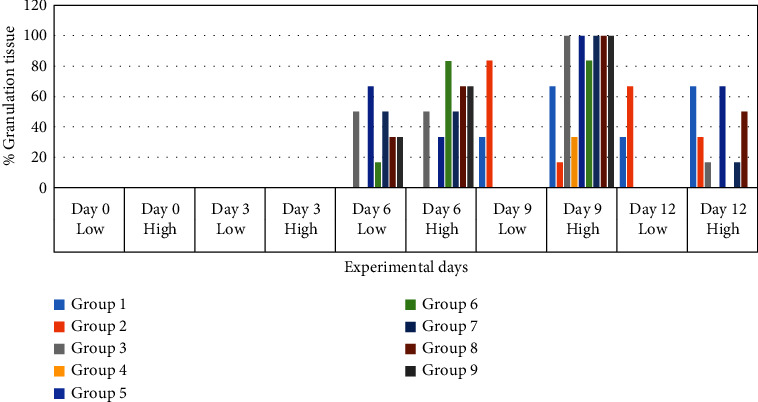
Wound granulation tissue (%) in experimental groups on days 3, 6 and 9, and 12 after wounding.

Compared to the nondiabetic control group and treatment groups, the diabetes control group's wound edge oedema was much worse (Figures 6 and 7). On day 3, it was noticeable in the SS, JS, NSK, TRB, and KAD control groups and completely absent in the HBP + M and DCH honey groups. Up to day 12 (*p* < 0.05), it was present in both the diabetes control group (50%) and the nondiabetic control group (33.3%). Compared to the diabetes control and SS, rats in the honey therapy groups healed their wounds with noticeably greater granulation tissue (*p* < 0.05). The pattern from days 6 to 9 was HBP > NSK > DCH > KAD > TRB > SS > JS (Figure 8).

### 3.5. Wound Bacterial Clearance

**Figure 9:**
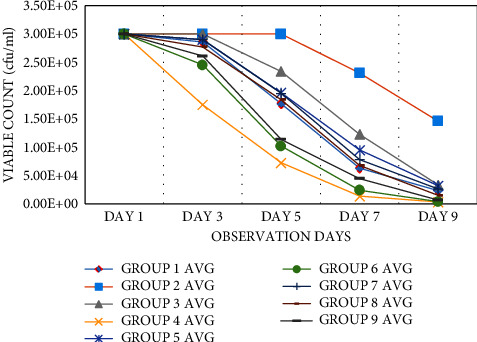
Microbial growth curve of *Staphylococcus aureus* postwound infliction on the different postwound treatment groups.

The rate of bacterial clearance (*Staphylococcus aureus*) in the SS group and the honey-treated groups was significantly higher than that in the diabetic untreated control group (positive control) (*p* < 0.05). The honey and SS treatment reduced the dense growth of *Staphylococcus aureus* on the culture plate on the different experimental days. Untreated diabetic wounds allowed dense growth of *Staphylococcus aureus* on the culture plates on days 1, 3, and 5 and then declined on days 7 and 9. On subsequent days 3, 5, 7, and 9, the honey treatment groups showed a significant reduction, allowing sparse *Staphylococcus aureus* on the culture plate. On day 9, the honey treatment groups and reference topical cream inhibited the growth of the *Staphylococcus aureus* colony, but the untreated wounds in the controls still showed dense growth (Figure 9).

### 3.6. Glucose Baseline

Table 4 shows the effect of alloxan on the glucose level before wound treatment (*p* < 0.05). The mean baseline of the fasting blood glucose in normal control rats was between 4.7 and 6.4 mmol/L. After induction, animals that had fasting blood glucose level > 200 mg/dL (11.1 mmol/L) were grouped as diabetic for treatment.

### 3.7. Effect of Treatments on Antioxidant Status and Oxidant of Wound Tissues

The groups determined the antioxidants and oxidant levels by measuring MDA and CAT levels (Table 5). Significance difference (*p* < 0.05) was observed in the CAT levels and MDA in HBP + M, JS, NSK, TRB, DCH honey, and 1% SS. MDA level was high in people with diabetes without treatment (14.55 ± 1.2) and with SS (11.62 ± 1.29). The oxidative stress marker MDA levels in the honey group exhibited a significant change compared to the positive control (the diabetic group with no treatment). Of the honey studied, JS, TRB, and KAD had (12.29 ± 2.1; 9.5 ± 3.15; 7.3 ± 0.28) MDA levels. MDA levels on day 17 were significantly lower in the honey groups and negative control group than in the positive control group (*p* = 0.037).

The least was observed in NSK and HBP + M (4.7 ± 6.65, 4.82 ± 0.41), while a significant difference (*P* < 0.05) was observed in CAT levels for HBP + M, TRB, and NSK (177.92 ± 107.8, 106.75 ± 7.18, 76.25 ± 21.57) when compared to the person with the diabetes-induced infection without treatment. No significant difference was observed in JS (20.34 ± 3.8) compared to diabetic and SS (Table 5) for CAT levels. The oxidative stress marker MDA levels in the honey group exhibited a significant change compared to the positive control (the diabetic group with no treatment). The level of CAT activity was the highest for HBP + M, and the differences between groups were not significantly notable in the treatment groups in comparison to the controls (*p*=0.117).

## 4. Discussion

A wound is said to occur when the typical structure and function of the outer tissue are altered, it can be healed by a biological process such as inflammatory response, proliferation, and remodelling to bring the injured tissue close to its original state. Diabetes can interfere with the normal healing process of the wound [28, 29]. Previous studies report that diabetes wounds are often infected with *Staphylococcus aureus*, methicillin-resistant *Staphylococcus aureus* (MRSA), and other resistant organisms [30, 31]. In recent studies, scientists have emphasized natural substances with antibacterial and antioxidant action with low toxicity to fight multidrug-resistant bacteria. Honey, moringa, and garlic, with their numerous beneficial bioactive components and medicinal benefits, have been studied in several ways. This forms the base for selecting honey and some plant extracts infused in honey as a potential wound-healing agent. Therefore, the present study evaluated the efficacy of honey in promoting wound healing in diabetes-induced wound infection in Wistar rats. In this study, we compared the use of a standard reference cream SS to the topical administration of various honey samples from different geographical locations and infused plant extracts on wound healing in diabetes-caused wound infection. The findings of this study showed that the different honey samples had varying efficacy. The low levels of aerobic mesophilic bacteria found in the samples indicate proper beekeeping methods and sanitary and storage conditions. All samples tested negative for faecal coliforms, indicating that good honey harvesting and conditioning techniques were followed, which agrees with data found by Laaroussi [32, 33]. Physiochemical parameters are usually analyzed in honey to ascertain the authenticity of the honey. It shows if the honey is adulterated or not and may be responsible for wound healing. The pH values of the honey samples were within the acidic range of 3.9 to 4.7, which is within the Codex Alimentarius [34] range. This is confirmatory to other studies irrespective of the geographical origin of the honey. The acidity of honey contributes to the acids produced in honey, particularly gluconic acid and minerals that aid in maintaining stability during storage and prevent microbial proliferation [35]. The acidity of honey also contributes to its antibacterial action and bacterial death. It makes it easier for oxygen to be released from the haemoglobin, which is necessary for the growth of new cells and the activation of white blood cells [36]. Honey can lower the pH of the wound, promoting tissue granulation and wound healing. A prior study measured the change in pH and the size of the ulcer to examine the effects of applying a manuka honey dressing on a nonhealing ulcer. A statistically significant decrease in the pH and size of the incision was observed [37]. The honey samples' mean moisture content (20%) fell within the range of good honey according to the Codex Alimentarius [34] specifications, except for HBP + G and TRB honey. Honey with less than 20% water content is hyperosmolar, which makes it difficult for microorganisms to thrive and survive. Because the water molecules are chemically linked to the sugar molecules, high osmolarity can hinder microbiological development and cause the death of organisms. This study's moisture content was higher than that in previous studies, which might be attributed to the harvesting time, storage conditions, environmental conditions, and processing methods [38–43]. No variations in the moisture obtained for the honey samples were statistically significant (*p* > 0.05). These findings show that this honey has high storage capacity. The ash content in pure honey reflects the overall amount of inorganic minerals. It should not be more than 0.6%. Variations were observed in the present study, which might be attributed to the various sources [44]. The fibre was not determined in the honey samples, while all the honey samples had greater protein levels and lipids than those indicated by international standards (0.34–1.72%). This might be associated with pollens, nectar, and bodies mixed during honey production and extraction [45].

The honey samples used in this study had degree Brix ranging from 72 to 83.4. According to reports, the Brix number is proportional to the honey's sugar amount. Brix readings will be more significant in honey with higher sugar content. Honey's acid and mineral content also contribute to the total soluble solids. This characteristic provides information about the honey's ripeness and nutritional worth [5, 46]. The fructose levels of all 11 samples of honey varied significantly. It was the primary sugar present in the samples. Low sucrose content implies that sucrose has completely been converted to glucose and fructose in mature honey. Honey creates a layer of protection against cross-contamination that contains enough hyperosmolar compounds to attract fluid into the region around the wound and create a viscous solution. As a result, a highly osmolar solution, such as the honey samples utilized in this investigation, can be used to treat wound infections brought on by diabetes. However, since honey's osmotic inhibition is eliminated when wound exudates dilute it, only pure honey is sufficient for suppressing bacteria development. According to the quantities of phenolic and flavonoids found in this study, the honey samples had antioxidant qualities that can aid in the healing of wounds. The botanical source heavily influences the amount and kind of phenolic chemicals found in honey. They increase circulation and prevent cell damage by inhibiting lipid peroxidation and enhancing the viability of collagen fibrils. They also aid wound healing by enhancing collagen deposition and their astringent and antibacterial qualities, which cause wound contraction and epithelialization [5]. The current investigation demonstrated that the test organism (*Staphylococcus aureus*) was susceptible to honey samples infused with plant extracts (moringa and garlic). The diverse zones of inhibition point to the distinct honey samples with the greatest DCH and lowest IMS having differing levels of effectiveness and different phytoconstituents. According to the findings, *Moringa oleifera* is responsible for the honey's bacteriostatic impact on the tested isolate. This shows a synergistic antibacterial effect evident in the increased zone of inhibition.

Wound contraction increased time-dependent in the honey samples, 1% SS, and control groups. These findings demonstrate that honey is a potent wound-healing agent capable of decreasing wound diameter as effectively as 1% SS. The different honey treatment groups (Group IV–Group IX) showed remarkable statistically significant wound closure ratios on days 3, 6, and 9 of treatment (*p* < 0.05). The early complete wound closure in the honey diabetic subgroup treatment showed a remarkable contraction equivalent to the SS treatments, which was related to the contraction of the wound and possibly enhanced fibroblastic response and reepithelization. These findings demonstrate that honey, when administered as a contracting agent, may reduce wound areas as effectively as SS, the topical medication employed as a standard. The study by Eyarefe et al. [18] showed that honey was compared favourably with amikacin, while the study by Subrahmanyam [47] compared the effectiveness of honey and 1% SS on wounds. Their findings showed that honey had a better healing and faster healing rate. The increased acute local inflammatory response was the primary cause of the prominence of wound surface exudation, oedema, and hyperemia in the groups, especially the untreated diabetic control for the first six days. Wound edge oedema and hyperemia are local indicators of acute inflammation to injury in clean wounds. If these symptoms persist after day three after injury, they may indicate wound infection and evidence of debridement difficulties [18]. This finding would explain the extended wound exudations between days 0 and 6 in the treatment groups, on day 9 in the diabetes control groups, and between days 0 and 12 in the untreated, SS, and JS groups. Acute wound wetness reflects the body's reaction to damage and is a regional indicator of infection. The wetness observed in the SS subgroup could be due to a high toxic MIC against *Staphylococcus aureus*, which could cause soft tissue reactions and an extended wound.

On the other hand, the diabetic honey treatment group's wound dryness may be related to hyperglycemia-induced dehydration brought on by frequent urine and insufficient fluid intake. The honey subgroup of the diabetic group had a significantly higher level of wound dryness, which highlights the unique wound-healing properties of honey. Between days 3 and 6, rats' wounds in the control groups and those in SS and JS honey had substantially higher wound edge oedema. However, there was reduced wound edge oedema in the diabetic group's honey subgroup, and there was a striking absence in the group receiving honey infused with moringa, moringa-infused honeybees smoothly apparent detritus without experiencing any discomfort. Due to honey's well-known antibacterial and anti-inflammatory qualities, monocytic cell activities, and efficient debridement process, the honey subgroup reduced wound edge oedema. All diabetes groups had delayed granulation tissue laydown. However, the subgroups of those who had honey and SS had excellent results. This finding may be explained by the fact that diabetes is linked to decreased and impaired levels of growth factor synthesis, angiogenesis responses, collagen accumulation, epidermal barrier function, and wound granulation tissue buildup. However, the honey subgroup displayed a gradual granulation tissue response, which may be explained by the honey's efficiency in clearing infections and promoting tissue granulation in wounds.

Compared to the positive control group and silver therapy (diabetic without treatment), honey therapy had lower oxidative stress marker MDA levels on day 17. Additionally, the rate of bacterial clearance in the honey therapy was directly correlated with the pace of wound healing. As a component of the cellular defence system, Nuclear Related Factor 2 (Nrf2) separates from its cytoplasmic repressor Keap 1 in response to oxidative stress and binds to cytoprotective genes that encode antioxidant enzymes in the nucleus [48]. Due to a lack of Nrf2, which impairs the antioxidant defence response and increases lipid peroxidation, the pace of wound healing in the diabetic control rats in our investigation (group II) was delayed. This was also observed in the SS and JS honey treatment groups. However, in groups IV, VI, VII, VIII, and IX, the nuclear Nrf2 was activated, followed by the antioxidant enzyme catalase (CAT) augmentation. The activities of the antioxidant enzyme (CAT) were reduced in the diabetic control rats (group II), SS, and group V. The group IV (honeybee infused with moringa) therapy produced a remarkable outcome. This outcome may be related to its antioxidant potential, as seen in the physicochemical parameter, which promotes wound healing by decreasing oxidative stress and raising antioxidant enzyme activities.

## 5. Conclusion

This study investigated the effect *Staphylococcus aureus* play in diabetic sores when left untreated, as well as how their actions may be capable of impairing the healing process and causing damage that is greater than anticipated. According to the findings of this study, honey is an effective alternative for treating wound infections since it possesses the qualities necessary to be used as a “standard to treat against diabetic wounds.” It is regarded as affordable and secure. Our study revealed that the inflammatory phase of wound healing began to decline early in the experimental groups with honey-dressed wounds. However, adding the plant extract moringa to the honey caused a miraculous recovery that included reduced wound exudation and hyperemia, fast contraction without scarring, and lack of inflammation (edge edema). The study showed that the phenolic content played a role in wound bacterial clearance, antioxidant activity, and antibacterial activity. The study concludes that honey from different zones in Nigeria possessed varying efficacy. Our findings suggest that honey infused with moringa is a suitable alternative option. It is acceptable due to its physicochemical properties, microbiological safety, antibacterial, antioxidant, and anti-inflammatory properties, and the promotion of tissue development and bacterial wound clearance found in this study. It is advantageous for managing wounds and being safe and economical. However, due to the study's findings that treatment response differs from host to host, depending on the peculiarities of the wound and the individual metabolism, care should be taken.

## Figures and Tables

**Table 1:** List of experimental groups.

Groups (*n* = 9)	Investigation
Group I	Negative control (nondiabetic with no treatment)
Group II	Positive control (diabetic with no treatment)
Group III	Diabetic (treatment: 1% silver sulfadiazine applied topically once daily for 17 days)
Group IV	Diabetic (treatment: HBP + M applied topically once daily for 17 days)
Group V	Diabetic (treatment: JS honey applied topically once daily for 17 days)
Group VI	Diabetic (treatment: NSK honey applied topically once daily for 17 days)
Group VII	Diabetic (treatment: TRB honey applied topically once daily for 17 days)
Group VIII	Diabetic (treatment: DCH honey applied topically once daily for 17 days)
Group IX	Diabetic (treatment: KAD honey applied topically once daily for 17 days)

**Table 2:** Microbial count of honey samples.

Honey samples	TVC (10^−2^ CFU·mL^−1^)	TCC (10^0^ CFU·mL^−1^)
KAD	1.3 × 10^4^	—
DCH	2.20 × 10^4^	—
HBP + G	1.0 × 10^4^	—
HBP + M	1.8 × 10^4^	—
ITEMS	1.05 × 10^4^	—
OBD	1.3 × 10^4^	—
KL	3.5 × 10^3^	—
JS	2.05 × 10^4^	—
TUB	1.45 × 10^4^	—
NSK	7.3 × 10^4^	—
HBP	3.5 × 10^3^	—

TVC: total viable count; TCC: total coliform count; KAD: Kaduna; DCH: Lagos; HBP + G: honeybee pure infused with garlic; HBP + M: honeybee infused with moringa; IMS: Imo state; OBD: Obudu; KL: US grade A honey; JS: Jos; TRB: Taraba; NSK: Nsukka; HBP: honeybee pure.

**Table 3:** Physiochemical parameters of honey samples.

Parameters	Legal standards (codex, 2001)	HBP	HBP + M	TUB	OBD	DCH	KAD	KL	HBP + G	JS	NSK	ITEMS
pH	3–5	4.7	4.5	4.5	4.4	4.3	4.5	4.6	4.4	4.3	3.9	4.1
Moisture (%)	≤21 g/100 g	16.76	19.43	21.05	15.04	12.16	16.05	17.82	27.43	18.17	17.48	17.01
Ash (%)	≤0.6 g/100	0.13	0.15	0.3	0.04	0.18	0.04	0.26	0.19	0.22	0.07	0.15
Fibre		ND	ND	ND	ND	ND	ND	ND	ND	ND	ND	ND
Crude protein (%)		1.72	0.45	0.44	0.34	1.27	0.59	0.7	0.97	0.41	0.58	1.14
Lipid (%)		1.23	2.21	2.01	2.8	2.2	2.84	0.2	1.03	1.26	2.08	2.23
TSS or degree Brix	≥65 g/100 g	81	72	76.5	78	83.4	78.6	79.8	75	79.2	78.6	78.6
Fructose (%)	No fixed limit	31.19	29.82	37.39	30.27	35.85	32.69	34.98	30.73	32.91	38.67	25.69
Glucose (%)	No fixed limit	32.03	35.95	25.41	30.75	30.14	32.21	26.82	30.66	30.33	25.41	39.1
Fructose + glucose (%)	>60	63.22	65.77	62.8	61.02	65.99	64.9	61.8	61.39	63.24	64.08	64.79
Sucrose (%)	≤5 g/100 g	1.78	1.82	2.13	1.79	1.85	2.12	1.78	1.73	1.94	1.79	1.9
Bioactive compounds												
Tannin (%)		6.88	4.86	4.02	1.5	3.54	2.35	3.35	6.68	1.79	1.55	2.1
Phenol												
(mg/100 g)		156.7	218.2	181.0	156.3	225.2	263.1	235.4	225.0	192.7	206.0	237.7
Flavonoid (%)		53.66	50.7	66.74	49.83	61.17	50.63	71.31	51.40	66.18	69.63	64.35

KAD: Kaduna; DCH: Lagos; HBP + G: honeybee pure infused with garlic; HBP + M: honeybee infused with moringa; IMS: Imo state; OBD: Obudu; KL: US grade A honey; JS: Jos; TRB: Taraba; NSK: Nsukka; HBP: honeybee pure; ND: not detected.

**Table 4:** Fasting blood sugar level of experimental groups before and after treatment.

Experimental groups	Glucose baseline	Glucose after
G1	5.72 ± 0.58	5.72 ± 0.58
G2	6.08 ± 0.53	17.58 ± 2.38
G3	5.98 ± 0.55	17.72 ± 2.87
G4	5.93 ± 0.74	16.1 ± 3.14
G5	5.62 ± 0.4	18 ± 3.7
G6	5.92 ± 0.59	19.12 ± 3.11
G7	6.17 ± 0.55	18.05 ± 4.82
G8	5.43 ± 0.63	20.55 ± 4.64
G9	5.45 ± 0.36	22.43 ± 4.98

**Table 5:** Summary of oxidant and antioxidant activity.

Experimental groups	CAT (U/ml)	MDA (mmol/ml)
Negative control	137.25 ± 64.7	5.13 ± 0.71
Positive control	66.08 ± 64.7	14.55 ± 1.2
SS	20.34 ± 14.38	11.62 ± 1.29
HBP + M	177.92 ± 107.83	4.82 ± 0.49
JS	20.34 ± 14.38	12.29 ± 2.1
NSK	76.25 ± 21.57	4.7 ± 6.65
TUB	106.75 ± 7.18	9.5 ± 3.15
DCH	30.5 ± 14.38	6.1 ± 3.54
KAD	61 ± 43.13	7.3 ± 0.28

Data are presented in values of mean (±standard deviation).

## Data Availability

All relevant data are included the paper.

## Abbreviations

CFU: Colony forming unit
SS: Silver sulfadiazine
MDA: Malondialdehyde
TBA: Thiobarbituric acid
COX: Cyclooxygenases
CAT: Catalase
TVC: Total viable count
TCC: Total coliform count
MRSA: Methicillin-resistant *Staphylococcus aureus.*
